# Penaeid Shrimp Chromosome Studies Entering the Post-Genomic Era

**DOI:** 10.3390/genes14112050

**Published:** 2023-11-07

**Authors:** Xiaojun Zhang, Jianhai Xiang, Jianbo Yuan, Fuhua Li

**Affiliations:** 1 CAS and Shandong Province Key Laboratory of Experimental Marine Biology, Institute of Oceanology, Chinese Academy of Sciences, Qingdao 266071, China; xjzhang@qdio.ac.cn (X.Z.); jhxiang@qdio.ac.cn (J.X.); yuanjb@qdio.ac.cn (J.Y.); 2 Key Laboratory of Breeding Biotechnology and Sustainable Aquaculture, Chinese Academy of Sciences, Wuhan 430072, China; 3 University of Chinese Academy of Sciences, Beijing 100049, China

**Keywords:** *Litopenaeus vannamei*, cytogenetics, genome, Hi-C, structure and function

## Abstract

Chromosome studies provide the foundation for comprehending inheritance, variation, systematics, and evolution. Penaeid shrimps are a group of crustaceans with great economic importance. Basic cytogenetic information obtained from these shrimps can be used to study their genome structure, chromosome relationships, chromosome variation, polyploidy manipulation, and breeding. The study of shrimp chromosomes experienced significant growth in the 1990s and has been closely linked to the progress of genome research since the application of next-generation sequencing technology. To date, the genome sequences of five penaeid shrimp species have been published. The availability of these genomes has ushered the study of shrimp chromosomes into the post-genomic era. Currently, research on shrimp cytogenetics not only involves chromosome counting and karyotyping, but also extends to investigating submicroscopic changes; exploring genome structure and regulation during various cell divisions; and contributing to the understanding of mechanisms related to growth, sexual control, stress resistance, and genome evolution. In this article, we provide an overview of the progress made in chromosome research on penaeid shrimp. We emphasize the mutual promotion between studies on chromosome structure and genome research and highlight the impact of chromosome-level assembly on studies of genome structure and function. Additionally, we summarize the emerging trends in post-genomic-era shrimp chromosome research.

## 1. Introduction

Decapods represent a significant group of crustaceans, including many species of high economic value, such as shrimp, crabs, lobsters, and crayfish. Among them, penaeid shrimps (Decapoda: Penaeidae) are the most commercially important species in fisheries and aquaculture [1]. The history of shrimp farming can be traced back to 1934, when Motosaku Fujinaga successfully obtained fertilized eggs and reared them to the mysis stage in Kuruma shrimp (*Marsupenaeus japonicus*) [2]. In the 1960s, researchers in aquaculture made a breakthrough in artificial larval rearing technology for shrimp. Shrimp farming has experienced explosive development since the 1980s, particularly in coastal areas of China and Southeast Asia, making it one of the most representative sectors in the marine aquaculture industry [3]. More than 60 countries are currently engaged in marine shrimp aquaculture, with a concentration in Asia and Latin America. According to the FAO (2022) [4], global shrimp aquaculture production exceeded 6.5 million tons in 2020. 

Despite the rapid development of the shrimp industry in recent decades, the occurrence of shrimp diseases has become increasingly serious due to germplasm degradation and environmental damage [1]. It is gradually being recognized that obtaining desirable traits in aquaculture, such as rapid growth, disease resistance, and controlled reproduction, and applying them to the industrial production of shrimp is unviable without the necessary biological and genetic knowledge [5]. Genetic improvement plays a crucial role in increasing aquaculture production and improving the quality of aquatic animals.

Chromosomes serve as carriers of eukaryotic genetic materials; they are the physical structures that contain the main portion of the genome. In addition, the uncoiling of chromosomes enables DNA synthesis and transcription to begin, and their three-dimensional structure affects gene expression, thereby playing a regulatory role in various functions. Therefore, understanding genetics through the study of chromosomes is essential. 

In 1948, the chromosome number of *M. japonicus* was shown to be 2n = 92 using the section method [6]. Subsequently, the chromosome numbers and karyotypes of dozens of economically important shrimp were reported. However, due to the lack of basic research, experimental difficulties, and inadequate investment, progress in shrimp chromosome research has been rather slow, basically limited to chromosome counting and karyotyping [7,8]. Only recently, with the advent of high-throughput genome sequencing, has a large volume of genome and transcriptome sequencing data been generated, and these data have driven innovation in chromosome-level assembly as well as chromosome structure and function studies [9,10]. In this review, we summarize the current status of shrimp chromosome research, highlight new findings, and propose future directions for cytogenetic and genome structure and function studies in penaeid shrimp. 

## 2. Chromosome Number, Morphology, and Evolution in Penaeid Shrimp

The lack of chromosomal data on these species can be attributed to the difficulty of finding tissues with a high number of dividing cells. After extensive exploration, chromosome analysis was carried out on samples of different developmental stages, including embryos, larvae, and adults, as well as from various tissues, such as the testis, antennal glands, midgut, and regenerated limbs [11]. So far, chromosome numbers of dozens of marine penaeid shrimp species have been reported (Table 1). Overall, most penaeid shrimps have a diploid (2n) chromosome number ranging from 88 to 92, with the modal chromosome number being 88. 

**Figure 1:**
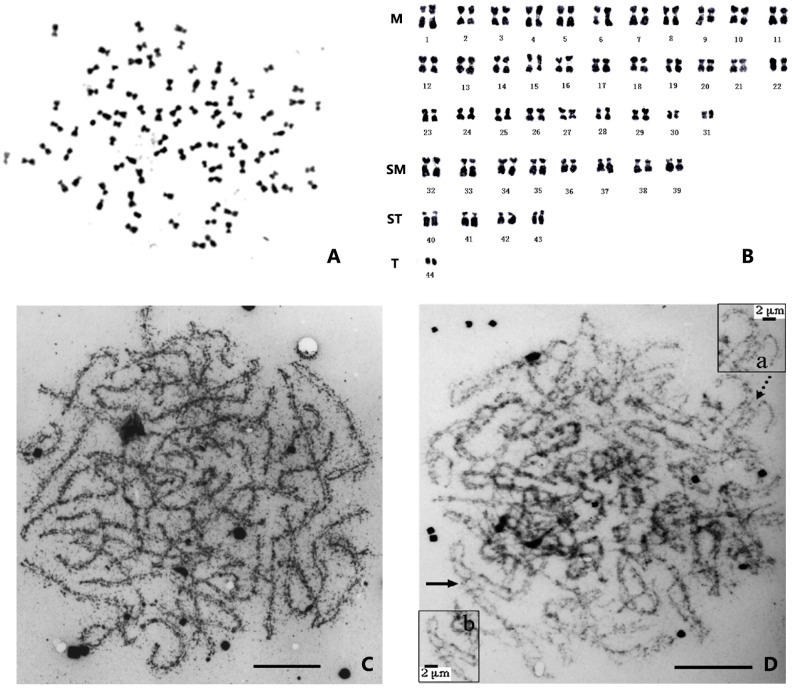
The chromosomes of the Chinese shrimp *Fenneropenaeus chinensis*. (**A**) Photomicrographs of a chromosome of *F. chinensis* [28]. (**B**) The karyotype of *F. chinensis* [29]. (**C**) Synaptonemal complex of *F. chinensis*. (**D**) Synapsis in triploid *F. chinensis* and details of irregular synaptic shapes in triploid [30]. Partner switches and arrows (**a**): totally paired trivalents with non-homologous region synapsis (**b**). Bar = 10 μm.

The metaphase division phases of shrimp chromosomes are generally small and similar, making them difficult to distinguish (Figure 1A). The morphology of chromosomes obtained from different tissues with different treatments is variable, leading to challenges in identifying corresponding homologous chromosomes and making accurate karyotyping almost impossible [12]. Preliminary karyotype analyses have shown that approximately half of the chromosomes are metacentric or submetacentric (M or SM), while the other half are subtelocentric or telocentric (ST or T). Among the latter, there are one or two pairs of smaller dot-like chromosomes, and their centromere position cannot be observed (Figure 1B).

**Table 1:** Number of chromosomes of different species of penaeid shrimp.

Species [13]	Alternative Species Name [14,15]	Number of Chromosomes	Karyotype	References
black tiger shrimp, *Penaeus monodon*	*Penaeus monodon*	2n = 88		[11]
		2n = 88	18A + 70B *	[16]
		2n = 88	16M + 20SM + 10ST + 42T	[17]
brown tiger shrimp, *Penaeus esculentus*	*Penaeus esculentus*	2n = 88		[18]
green tiger shrimp, *Penaeus semisulcatus*	*Penaeus semisulcatus*	2n = 90		[11]
		2n = 90		[7]
brown shrimp, *Farfantepenaeus aztecus*	*Penaeus aztecus*	2n = 88		[19]
		2n = 88	18 M + 18 SM + 52A **	[20]
		2n = 88		[21]
		2n = 88		[11]
Pacific brown shrimp, *Farfantepenaeus californiensis*	*Penaeus californiensis*	2n = 92	14M + 78ST	[22]
		2n = 88	4M + l0SM + 52ST + 22T	[23]
pink shrimp, *Farfantepenaeus duorarum*	*Penaeus duorarum*	2n = 88		[19]
		2n = 88		[21]
		2n = 88		[11]
Chinese shrimp, *Fenneropenaeus chinensis*	*Penaeus chinensis*	2n = 88	26A + 15B + 3C ***	[24]
		2n = 88	54M + 20(M, SM) + 10SM + 4(SM, ST)	[25]
Indian white shrimp, *Fenneropenaeus indicus*	*Penaeus indicus*	2n = 88	27M + 13SM + 4ST	[12]
banana shrimp, *Fenneropenaeus merguiensis*	*Penaeus merguiensis*	2n = 88		[18]
		2n = 88	21(M, SM) + 23(T, A **)	[8]
redtail shrimp, *Fenneropenaeus penicillatus*	*Penaeus penicillatus*	2n = 88		[11]
Western white shrimp, *Litopenaeus occidentalis*	*Penaeus occidentalis*	2n = 92	14M + 78ST	[22]
white shrimp, *Litopenaeus setiferus*	*Penaeus setiferus*	2n = 90		[19]
		2n = 90		[21]
		2n = 88		[11]
blue shrimp, *Litopenaeus stylirostris*	*Penaeus stylirostris*	2n = 88		[26]
		2n = 92	14M + 78ST	[22]
Pacific white shrimp, *Litopenaeus vannamei*	*Penaeus vannamei*	2n = 92	14M + 78ST	[22]
		2n = 88		[21]
		2n = 88	4M + l0SM + 56ST + 18T	[23]
		2n = 88		[26]
Kuruma shrimp, *Marsupenaeus japonicus*	*Penaeus japonicus*	2n = 92		[6]
		2n = 86		[27]
		2n = 86		[11]

* Sorting the chromosomes according to size, those with an obvious X-type were considered as metacentric and submetacentric chromosomes and classified as group A; others were classified as group B chromosomes. ** A represents an acrocentric chromosome. *** An A-type chromosome is shaped like an “X”, a B-type chromosome is shaped like an inverted “Y”, and a C-type chromosome is dot-like.

Penaeid shrimp possess some small (dot-like) chromosomes, but it remains uncertain whether these chromosomes are microchromosomes or B-chromosomes. Microchromosomes, which are found in certain vertebrate species, such as birds and fish, typically exhibit a higher GC content, greater gene density, lower density of repetitive sequences, and higher recombination rates compared to large chromosomes [31]. B-chromosomes were originally thought to have little or no effect on the biology or fitness of organisms. However, research has shown that they can be important players in the genome, with a significant impact on evolution [32]. Although these specific types of chromosomes are not clearly present in crustaceans, small dot-like chromosomes are widespread. Varied numbers of small chromosomes can be found in many Caridea species, with crabs exhibiting the highest prevalence of small chromosomes [33]. Therefore, studying the origin, structure, evolution, and function of these small chromosomes would be highly valuable.

The origin and evolution of chromosomes have long eluded scientists. Recent studies have indicated that metazoans first appeared more than 600 million years ago. It is still possible to identify segments of the ancestral chromosomes that have remained intact over that time, but the order of the genes in the chromosomes is often disrupted [34]. Despite these disruptions, the genome itself has shown stability over a long period of evolution. These findings suggest a startling hypothesis that the mixing of these genomes over the last 500 million years may be associated with the emergence and diversity of new species. However, the ancestral chromosomes of pancrustaceans that appeared in the early Cambrian period have not been studied. Comparative analyses of shrimp genomes at the chromosomal level with other metazoan genomes can provide insights into the fusing and recombining of shrimp chromosomes. Additionally, by understanding and controlling the rules of chromosomal changes, researchers can predict such changes in the genomes of other species that have not yet been sequenced.

Despite the continuous development of modern cytogenetic techniques, shrimp chromosome research has not made corresponding progress using new techniques, such as electron microscopy, laser scanning microscopy, multiple fluorescent in situ hybridization (M-FISH), spectral karyotyping (SKY), laser microdissection (LM), and optical genome mapping (OGM), as reported in model organisms, livestock, poultry, and crops [35,36,37]. However, in the field of shrimp genomics, some significant breakthroughs have been made. Therefore, it is important for us to combine genomics methods with novel cytogenetic techniques in order to make new discoveries regarding shrimp chromosomes.

## 3. Ploidy and Chromosome Manipulation in Penaeid Shrimp

Chromosome studies contribute to a better understanding of the shrimp genome in various aspects. For instance, karyotypes can help to identify potential hyperploidy and aneuploidy events, while molecular cytogenetics and epigenetic markers aid in the comprehension of adaptive genome evolution [38]. A previous study suggested that changes in decapod chromosome numbers might be attributed to polyploidization [39]. However, due to the morphological similarity of shrimp chromosomes, it remains challenging to determine whether polyploidization has occurred. In our studies of the *F. chinensis* and *L. vannamei* genomes, despite the large genome size and the presence of numerous repetitive sequences, no evidence of genome duplication was found based on a series of different approaches [9].

Chromosome set manipulation is a popular research topic in shrimp genetics, as it provides rapid methods for gonadal sterilization, sex control, improvement of the hybridization survival rate, and cloning efficiency [40]. These techniques involve manipulating the chromosomes to induce polyploid (triploid and tetraploid) and uniparental inheritance (gynogenesis and androgenesis) in animals. The application of triploid technology in shrimp is believed to increase their size and accelerate their growth rate, and to help to improve the yield and quality of shrimp aquaculture [41,42,43]. Females are generally larger than males in all penaeid shrimp species, making the production of all-female shrimp a matter of potential commercial importance [40,44,45]. Since the demonstration of chromosome manipulation feasibility in shrimp in 1991 [46], numerous studies have reported the successful induction of polyploidy. Triploid embryos, larvae, and adults have been produced in several commercial shrimp species, including *F. chinensis*, *M. japonicus*, *L. vannamei*, *P. monodon*, and *F. indicus* [40,44,45,47,48].

The induction of polyploidy in tropical shrimps such as *L. vannamei* and *P. monodon* is often challenging due to their rapid embryonic development and the short induction window. However, in *F. chinensis* and *M. japonicus*, which survive in colder environments, it is possible to achieve a high triploidy induction rate, even reaching ~100% [47,49]. When attempting to induce polyploidy in *L. vannamei* through cold shock, it was found that abnormalities and chimerism in the embryos could lead to varying proportions of diploids or mosaic individuals [50]. No significant difference in triploid rates was observed between the embryonic and larval stages, suggesting that triploid embryos can hatch like diploid embryos [47]. A study on *M. japonicus* also found no significant difference (*p* > 0.05) in survival rates between the embryonic and larval stages for triploids, while the average survival of diploids was typically highest in the control group [49].

An analysis of adults from a polyploid induction has shown that the sex of triploid shrimp is female-preferred; for example, 80% were females in *F. chinensis* [51] and 100% were females in *M. japonicus* [44]. Additionally, female/male ratios of 16:1 and 2:1 have been reported in *M. japonicas* and *P. monodon*, respectively [49,52]. However, in a triploid induction experiment on *P. monodon*, triploidy was found not to skew the sex ratio for females, resulting in a female/male ratio of 1:1.625 [53]. These findings have far-reaching scientific implications for the mechanisms of sex determination and differentiation in shrimp, as well as for the development of monosex cultures [54].

Triploids are generally sterile. The degree of testicular development and sperm counts of triploid *F. chinensis* are significantly lower than those of diploids [51], and while a few sperm-like cells can be observed in the vas deferens of male triploids, these cells are larger than those of diploid sperm [40]. In addition, the ovary development of triploid shrimp is significantly delayed, and the gonadosomatic index (GSI) of triploids is also lower than that of diploids. The spermatocytes of triploid *F. chinensis* have very complex synaptic configurations; the unpaired axes are tangled, and in the late pachytene stage, the connection of all three homologues occurs in the same region (Figure 1C,D); this synaptic behavior may be the key to the production of 3n sperm [30]. Meanwhile, suppression subtractive hybridization (SSH) has been performed using diploid and triploid ovaries, and differential genes have been identified, including *PCNA* (proliferating cell nuclear antigen), *CAS*/*CSE1* (cellular apoptosis susceptibility/chromosome segregation 1), and *SSRF* (spermatogonial stem cell renewal factor). These findings indicate that the gene regulatory network in triploid ovaries is affected by chromosome triplication [55].

Compared to triploids, there has been little research on tetraploid induction and other chromosomal manipulation in shrimp. Tetraploids can be used as male parents for all-triploid industrial breeding. However, in most cases, the induction and survival rates of tetraploids are low for penaeid shrimp [45,56,57,58]. In addition, chromosome manipulation can induce either gynogenesis or androgenesis. The production of gynogenetic individuals is of particular interest to geneticists and breeders because it can lead to high levels of inbreeding in a generation, as in some fishes. Moreover, gynogenesis can facilitate the breeding of all-female populations, enhancing our understanding of the mechanisms of sex determination and epigenetic regulation in these species. However, no successful acquisition of gynogenetic or androgeneic shrimp has been reported.

## 4. Gene Mapping

In order to cultivate shrimp strains that are disease-resistant and grow quickly under varying environmental conditions (such as low salinity, low temperature, closed recirculation systems, high pollutant concentrations, etc.), identifying the markers and genes associated with these traits is the first step [59]. To achieve this objective, the construction of a genetic map of shrimp began very early on [60]. Over the years, with advances in marker development and mapping technology, markers such as restriction fragment length polymorphism (RFLP), randomly amplified polymorphic DNA (RAPD), amplified fragment length polymorphisms (AFLPs), simple sequence repeat (SSR), and single-nucleotide polymorphisms (SNPs) have gradually been used to improve the accuracy and density of the shrimp genetic map. Today, high-throughput sequencing technology has rapidly advanced the genetic map of shrimp. Over the past twenty years, genetic maps with hundreds and thousands of markers have been constructed in economic shrimp species such as *L. vannamei*, *P. monodon*, *F. chinensis*, and *M. japonicus*. Their marker density on chromosome linkage groups has also increased (average inter-marker distances of 0.7, 0.39, 0.9, and 0.41 cM, respectively) [61,62,63,64,65,66]. Quantitative trait loci (QTLs) associated with some important economic traits, such as sex, body length, body weight, and disease resistance, have been mapped. Although these maps are useful for shrimp genetics and breeding, more accurate genome annotations and highly continuous and complete reference genomes are needed for comprehensive comparative genomics analysis to identify key genes or structural variations to guide breeding.

Unlike genetic maps based on genetic linkage principles, physical mapping aims to determine the order and spacing of genes or genetic markers on chromosomes. Physical genome maps can be divided into different types: chromosome maps, restriction enzyme digestion maps, contig maps, and DNA sequence maps. The simplest map is the chromosome-banding map, while the most elaborate one is the whole-genome DNA sequence map. However, due to the difficulty of preparing chromosomes, as well as the small size and similarity of chromosome morphologies, there have been few studies on chromosome banding. Only a limited number of chromosome localizations have been reported using the low-resolution FISH technique, such as 5S rDNA [67] and telomere sequence [1,68]. Even with the completion of whole-genome sequencing in shrimp, identifying all or a certain number of chromosomes still presents a significant challenge. Recently, the introduction of third-generation sequencing and chromosome conformation capture technologies has greatly improved the quality of physical map construction, and has facilitated studies such as genome assembly and genome structural variation. Genetic mapping, in recent years, has also enhanced the localization of specific genes and the study of structural variation, especially regarding the localization of sex-determining segments [69,70]. Currently, genome-wide association studies (GWAS) have been conducted based on chromosome-level genome reference mapping to identify genes associated with economically relevant traits such as growth, sex, disease resistance, and environmental adaptation. Resequencing and selection characterization have also confirmed that genes linked to growth and disease resistance are indeed selected for in artificial breeding [71].

## 5. Whole Genome Assembly and Structure Analysis of Shrimp

High-quality chromosome-level reference genomes are crucial for understanding the molecular mechanisms of important biological processes [72]. The shrimp genome presents a long-standing challenge in research with respect to aquatic animal genomes due to its large number of chromosomes, high heterozygosity, and complex repetitive elements. The analysis of the entire shrimp genome has always been a major concern in this field [73].

### 5.1. Genome Assembly at Chromosome Level

The high percentage of repetitive sequences and the difficulty of DNA manipulation due to the large amount of mucopolysaccharides and phosphatases were the main reasons for the delay in sequencing and assembling the shrimp genome until 2019 [73,74]. Before the application of third-generation long-reads sequencing technology, many attempts to assemble the shrimp genome with short sequencing reads resulted in highly fragmented assemblies, illustrating the repeatability and complexity of the shrimp genome [1,74,75,76]. Long-reads sequencing using the PacBio or Nanopore platforms to sequence shrimp DNA has effectively solved the problem of fragmented genome assembly. Additionally, Hi-C (high-through chromosome conformation capture) technology effectively divides, sorts, and orients the genome contig sequences into chromosomes, thereby improving the continuity of genome assembly. These two methodological improvements are crucial for obtaining a complete and continuous reference genome, and for accurately predicting duplicated elements and protein coding gene models in the genome [74,76]. By August 2023, the complete genome maps of five penaeid shrimp species had been published successfully, with some reaching the level of chromosome assembly [1,9,10,77,78,79] (Table 2).

The *L. vannamei* genome is approximately 2.45 Gb in size [1]. The economically important shrimp genome was obtained in 2019 by utilizing different sequencing techniques including Illumina, PacBio long-reads, and BAC end sequencing, along with multiple attempts with different genome assembly programs [1]. Ultimately, the WTDBG [81] program produced the most optimal assembly of the genome by using long-reads as a framework, correcting with Illumina clean reads, and scaffolding with mate-pire libraries and BAC end sequences. This assembly had a contig N50 of 57.65 Kb and a scaffold N50 of 605.56 Kb. Of the 4683 scaffolds, 3275 were anchored to 44 pseudochromosomes, representing 87.34% of the genome assembly size. The *L. vannamei* genome was the first high-quality reference genome for penaeid shrimp, and since then, the strategy has been used to sequence most crustacean genomes, including various shrimp, crayfish, crabs, krill, isopods, and amphipods [10,71,79,82].

**Figure 2:**
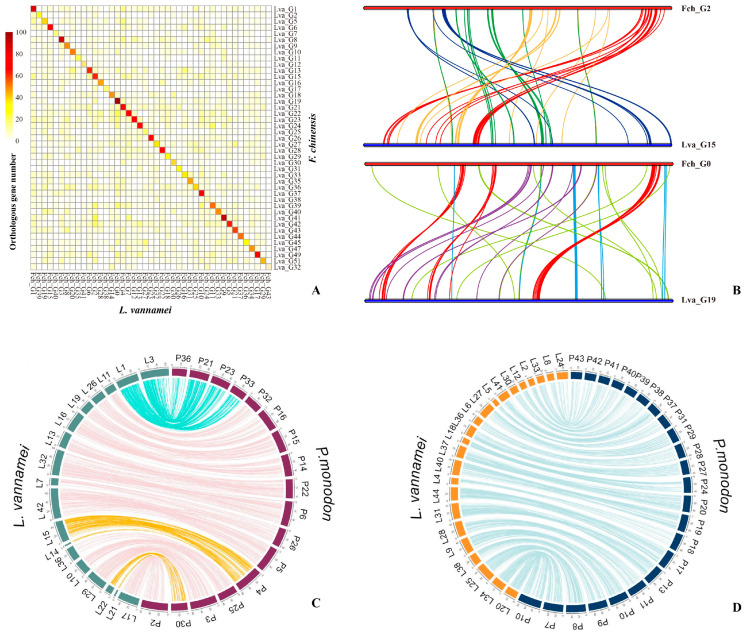
Collinearity between *F. chinensis*, *P. monodon*, and *L. vannamei* pseudochromosomes. (**A**) Heatmap of the orthologous gene numbers in each pair of chromosomes from *F. chinensis* and *L. vannamei* genomes [9]. (**B**) Intrachromosomal rearrangement between the homologous chromosomes of *F. chinensis* and *L. vannamei* [9]. (**C**) Lines link the synteny block with sequence coverage > 1 kb and identity >90%. “P” represents the *P. monodon*, and “L” represents the *L. vannamei* [10]. (**D**) Diagrams showing collinearity between *P. monodon* and *L. vannamei* where the syntenic relationship between pseudochromosomes is one-to-one [10]. (**C**) and (**D**) reused from publication by [10].

Subsequently, the genome of the Chinese native shrimp *F. chinensis* was sequenced [9]; it has a smaller genome size (1.88 Gb) than that of *L. vannamei*. The genome assembly of *F. chinensis* is comparable to that of *L. vannamei*, as the sequencing data cover more than half of the *L. vannamei* genome. This suggests the presence of a significant proportion of homologous sequences between the two shrimp species. However, synteny analysis revealed that, although the distribution of orthologous genes in the two species showed a one-to-one correspondence between their chromosomes (Figure 2A), the synteny of the distribution of orthologous genes within the chromosomes was poor (Figure 2B). This indicated that a significant number of rearrangements had occurred within the chromosomes. Further analysis revealed that the genomes of Penaeidae shrimp contained an extremely high SSR content (>20%). The explosive expansion of SSRs mainly originates from the carriage of transposable elements, leading to the hypothesis that SSRs are key factors causing a large number of genomic rearrangements. Moreover, it has been found that SSRs are enriched in gene regulatory regions and are involved in gene expression in the key osmotic regulation pathways [9]. These differences in gene expression patterns may be an important factor contributing to the variations in osmotic regulation between the two shrimp species, suggesting that SSRs play a crucial role in the adaptive evolution of shrimp [9,83]. Another version of the *F. chinensis* genome assembly also obtained a high-continuity genome. However, it showed a low correlation between contigs on the last pseudochromosome in the HiC heatmap, suggesting that there may be only 43 pairs of chromosomes in the somatic cells of *F. chinensis*, similar to *M. japonicus* [77].

In 2021, a combination of long-reads from PacBio, along with long-range Chicago and Hi-C technologies, were utilized to successfully assemble a 2.39 Gb *P. monodon* genome [10]. The assembled contig N50 reached 79 kb and comprised 44 pseudochromosomes, covering 82.9% of the total assembled sequence. The high continuity of the assembly at the chromosome level enabled homologous chromosome analyses between *P. monodon* and *L. vannamei*. The conserved orthologous gene sections between the black tiger shrimp and the Pacific white shrimp are shown in Figure 2C,D. The distribution of paralogous gene pairs showed a one-to-one correspondence between the 15 chromosomes (linkage groups). There was also a one-to-two correspondence between chromosomes 4 and 30 of *P. monodon* and chromosomes 14, 15, 21, and 22 of *L. vannamei* [21]. This suggests that some chromosomes derived from the common ancestor of the two species may have been split into two smaller chromosomes in *L. vannamei*, while remaining unsplit in *P. monodon*. Another *P. monodon* genome from Australia showed minimal variation from the genome of *P. monodon* from Thailand, except for some fragments of endogenous viral elements showing differences in their order and arrangement [76].

In *M. japonicus*, two separate teams have constructed genome-wide maps [79,80]. The Hi-C data anchored 18,019 contigs onto 42 pseudochromosomes [79]. The number of chromosomes in *M. japonicus* (2n = 86) is one pair less than that found in most penaeid shrimps. It is generally believed that the reduction in chromosome number is mainly due to chromosome fusion or loss. However, which two chromosomes in *M. japonicus* have undergone fusion, or which chromosome has been lost, need to be determined by comparing them with more complete and accurate genomes from different penaeid shrimps.

In 2022, a "superior contiguous" genome assembly of *F. indicus* was published with contig N50 1.4 Mb and scaffold N50 34.4 Mb [78]. This assembly met the criteria for animal genomes, which require contig N50 and scaffold N50 greater than 1 Mb and 10 Mb, respectively [84]. Compared to other shrimp genomes, the chromosome-scale assemblies provided for *F. indicus* have fewer gaps [78].

However, the majority of shrimp genomes remain incomplete due to the large number of repetitive sequences and their large genome sizes; therefore, there is a requirement for higher-quality shrimp genome information at the chromosomal level. At present, many scaffolds do not align with chromosomes in a one-to-one correspondence, and achieving a BUSCO (benchmarking universal single-copy orthologs) completeness score exceeding 95% remains challenging. Fortunately, researchers have been diligently working on enhancing the quality of shrimp genomes, bringing us closer to accomplishing these objectives.

### 5.2. Shrimp Genome Structure Analyses

Analysis of the genome sequences has revealed that penaeid shrimp do not appear to undergo lineage-specific whole-genome duplication, unlike chelicerates (e.g., spiders and horseshoe crabs) and vertebrates such as fish [74]. The presence of a high proportion (>50%, much higher in K-mer analysis) of repetitive sequences in the shrimp genomes may be associated with their deeper chromosomal condensation. Additionally, the rapid expansion of SSRs in intergenic regions and the abundance of repetitive elements may contribute to the formation of larger genomes and facilitate environmental adaptation [9]. It has also been suggested that the expansion of histone-encoding genes may contribute to the maintenance of larger chromosomes, which is correlated with the global compacting mechanism of chromosomes during mitosis [85,86]. Indeed, an expansion of histone genes has been found in the genomes of *L. vannamei* [1].

It is particularly noteworthy that the shrimp genome has, by far, the highest proportion of SSRs (19.15–43.13%) (Table 2) among the animal genomes that have been sequenced. The high SSR content is an important feature of the shrimp genome, and its origin and role in the plasticity of the shrimp genome have been partially elucidated [9]. However, its deeper mechanisms and consequences remain to be explored, such as the occurrence of surprising structural rearrangements within shrimp chromosomes. Chromosome-level assembly can enable the in-depth study of the structure and functions of repetitive elements. Although there is a lack of appropriate platforms for studying the biological functions of repetitive sequences, they may be associated with important functions found in other organisms. For instance, LINE/I is a significant component in penaeid shrimp genomes, and in humans, LINE/I has been discovered to play a crucial role in mobilizing DNA to new locations [87]. Considering that the diversity of repetitive sequences is believed to have an essential role in animals’ environmental adaptation [88], further exploration into the functions of repetitive elements in shrimp will help to enhance our understanding of shrimp biology.

## 6. Sex Chromosomes of Penaeid Shrimp

The sexual dimorphism in shrimp is quite prominent. In the breeding process, the body weight of mature female shrimp is significantly higher than that of male shrimp. Therefore, monosex cultures can have a significant impact on economic efficiency, as has been achieved in the freshwater shrimp species *Macrobrachium rosenbergii*. With the rapid development of shrimp culture, studies on sex determination and differentiation are becoming increasingly important [89,90].

The sex chromosomes of shrimp have been a popular topic, even though none of the available karyotypes have been able to distinguish between sex chromosomes and autosomes, and no confirmed sex-determining genes have been identified in any shrimp [91]. Many clues related to sex have been discovered in triploid studies on shrimp. If the females are heterogamous, then the triploid offspring will possess genotypes (WWZ), (WZZ), or (ZZZ) [44,51,92,93], which should be associated with a high proportion of females in the triploid shrimp population. The triploids of *F. chinensis* and *M. japonicus* have been identified as having a female preference, suggesting that females have heterogametic (ZW) sex determination in penaeid shrimps [44,51,92]. Genetic maps also show that shrimp are female heterogamety and possess a stable mechanism of sexual inheritance [61,62,63,64,65,66].

Sexual differentiation is the result of a cascade of gene expression events triggered by a sex-determining gene located on the sex chromosome [94]. To date, although the genomic information on five economically important penaeid shrimp species has been well annotated, successful mapping of sex-determining genes at the chromosome level has not yet been achieved. The sex-determining region of *L. vannamei* has been found to contain numerous repetitive sequences and exhibit a highly complex structure. Combining genome sequencing and genetic mapping, the position of this sex-determining region was ultimately located on the LG18 linkage group, and a sex-linked marker was obtained [95]. Subsequently, based on the above finding, a sequence from the sex-determining region was acquired and turned out to display differences in the copy number amplification and sequence length between males and females [77]. Meanwhile, five sex-linked markers were identified through a combination of resequencing data and GWAS analysis [77]. Additionally, three differentially expressed gonadal genes were found in scaffold 2550 of *F. chinensis*, and four were found in scaffold 3683. All of these genes exhibited high expression levels in males, with two encoding PBRA1 (peripheral-type benzodiazepine receptor-associated protein 1), two encoding TSPOAP1 (TSPO-associated protein 1), and the latter involved in steroid hormone biosynthesis. It is hypothesized that these highly expressed male genes are associated with sex-specific physiological mechanisms related to reproductive function in shrimp, and they may play a role in the sex differentiation process [96].

Shrimp genome projects generally select an adult male for genome sequencing and assembly, thereby omitting the chromosomal information of female shrimp. However, when comparing resequencing data from male and female *F. chinensis* populations to the reference genome, it was found that there were no significant differences in the rate of comparison or coverage of the genome. This suggests that there is less differentiation in their sex chromosomes [96]. Heteromorphic sex chromosomes have been reported to result from the suppression of chromosomal recombination during sex chromosome formation due to selective pressures acting on the sex-determining region and sexually antagonistic mutations in its vicinity [97,98]. However, in many species, the suppression of recombination near the sex-determining locus has not yet spread throughout the sex chromosomes, resulting in a limited level of differentiation rather than heteromorphic sex chromosomes [97,98].

Despite numerous efforts, the sex chromosomes and sex-determining genes of shrimp remain largely uncertain. It has been suggested that Cephalocarida and Remipedia, as the two most primitive clades of crustaceans, represent the ancestral sex system of crustaceans, i.e., simultaneous hermaphroditism [99,100]. In this case, there may be a mechanism by which sex chromosome differentiation is suppressed, particularly in Penaeidae species that retain more ancestral characteristics [101]. To date, all ideas regarding penaeid shrimp sex chromosomes have been hypotheses that require further validation. With the completion of the five genomes, high-quality reference sequences are available as a genetic foundation for conducting additional research on the mechanism of sex determination in penaeid shrimp. With advancements in in situ hybridization technology and powerful gene editing tools, the mystery surrounding the existence of sex chromosomes and the mode of sex determination in shrimp is expected to be resolved soon.

## 7. Chromosome Structure and Gene Regulation

Eukaryotic chromosomes are highly condensed and can adopt various conformations within the nucleus. Chromatin contacts can reflect both spatial and linear distances between different DNA segments. Furthermore, the three-dimensional organization of the genome is crucial for regulating gene expression. However, due to the large size of the genome and the vast amount of information contained in paired data, the cost of obtaining high-resolution whole-genome chromatin contact maps is prohibitive. Thanks to high-throughput sequencing technology, methods based on chromosome conformation capture (such as Hi-C, 3C, 4C) have been widely used for obtaining chromatin contacts. The HiC map, or chromosome conformation map, can show the three-dimensional organization of chromosomes within the nucleus. It provides information on how different regions of the genome physically interact with each other, revealing insights into chromatin folding and the spatial organization of chromosomes. Moreover, the frequency of interactions between chromatins is higher, resulting in a higher marker density. This can be utilized to assemble scaffolds or contigs, enabling the localization of over 90% of genome sequences on chromosomes. In shrimp genome projects, Hi-C-assisted assembly has played a crucial role in achieving chromosome-level assembly [9,10,76,77,79].

Hi-C analysis can capture information about the spatial interaction between different gene loci and identify DNA elements that regulate genes. It utilizes the spatial relationships in the entirety of chromatin DNA throughout the genome, achieving a comprehensive analysis of the three-dimensional structure of the entire genome [102]. Initially, Hi-C was unable to distinguish sister chromatids based on their sequence similarity. However, more recently, ultra-long-segment sequencing has made it possible to distinguish sister chromatids. This, in combination with chromosome conformation capture technology, can provide a detailed understanding of the internal organization of chromosomes.

Increasing evidence has shown that the formation of high-level chromatin structures is not random, but rather a crucial factor in regulating gene expression [103]. In 2022, a high-density genome-wide chromatin contact reference map was constructed using a meta-analytic approach integrating Hi-C experiments from 3600 humans, 6700 mice, and 500 flies. It displayed the unique ability of meta-Hi-C chromatin contact maps to capture cis- and trans-functional chromatin contacts, creating an integrated chromatin interaction network that can predict co-expression, eQTLs, and cross-species relationships [104]. Additionally, in lampbrush chromosome studies, researchers have analyzed the correspondence between genome-wide data and microscopic visualization [105,106]. These studies have revealed that genes may be far apart on the chromosome, but looping can bring them physically close together and allow them to share biological mechanisms.

In terms of the chromosome structure involved in the regulation of gene expression, chromatin accessibility has been shown to reflect the binding state of regulatory factors to open chromatin and is closely related to transcriptional regulation. The assay for transposase-accessible chromatin sequencing (ATAC-seq) is now the preferred technique for studying chromatin accessibility, and can be used in conjunction with other methods to screen for specific regulatory factors of interest. In a study of the genome structure of *L. vannamei* and *F. chinensis*, SSRs were found to be enriched in accessible chromatin regions and involved in regulating gene expression in key osmotic pathways, specifically glycine, serine, and threonine metabolism [9]. The composition of SSRs located within introns or UTRs showed significant differences in osmotic orthologous genes, and these differences contribute to the variations in osmotic regulation ability between *L. vannamei* and *F. chinensis* [18]. These investigations represent a crucial advancement in the study of genomic plasticity and evolution of environmental adaptation in shrimp during the post-genomic era. High-throughput sequencing and bioinformatics approaches, combined with microscopic visualization techniques, have become vital tools for chromosome studies, and these new directions are particularly exciting. As technology continues to advance, a strong correlation between the spatial structure of the shrimp genome and transcriptional regulation will be established, facilitating our comprehensive understanding of shrimp chromosome structure, regulatory mechanisms, and biological processes.

## 8. Concluding Remarks

Through chromosome-level genome assembly, the bottleneck in genetic information caused by the lack of genomic resources in shrimp has been largely resolved. This breakthrough has enabled the exploration of the genetic basis of shrimp and facilitates genome-assisted breeding. It is foreseeable that in the future, most genomes of penaeid shrimp will be sequenced, and the quality of genome assemblies will continue to improve. The next challenge lies in effectively utilizing genomic data to conduct large-scale studies on genetic structural variation, genome evolution, and karyotype dynamics in shrimp. Chromosome imaging, chromosome rearrangement, meiotic modifications, and the origin and evolution of karyotype-specific features (including sex chromosomes and heterochromatin distribution), as well as the higher structure and regulation of chromosomes, are likely to be the topics of future research on shrimp chromosomes. By integrating data from all cytogenomic approaches, researchers can obtain a comprehensive understanding of chromosomal characteristics in this economically important crustacean lineage. This integration will provide insights into chromosome biology, genome evolution, and organization. It will further promote the development of fundamental sciences such as comparative genomics, evolution, systematics, and ecosystem adaptation in shrimp. At the same time, genomic knowledge will be applied to the practices of aquaculture and fisheries, including genetic resource management and selective breeding, in order to foster the sustainable development of aquaculture and fisheries.

## Figures and Tables

**Table 2:** Summary of assembly statistics for the sequenced penaeid shrimps.

Species	Chr Level	Assembled Genome Size (Gb)	Contig N50 (Kb)	Scaffold N50 (Kb)	Gene Number	Repeat/SSR Content (%)	Sequencing Platform	Publication Year	Reference
*L. vannamei*	N	1.63	57.65	606	25,596	51.7/23.93	Illumina + PacBio + HiC	2019	[1]
*F. chinensis*	Y	1.58	58.996	28,916	26,343	48.58/19.5	Illumina + PacBio + HiC	2021	[9]
*F. chinensis*	Y	1.47	472.84	36,871	25,026	57.73/-	Illumina + PacBio + HiC	2021	[77]
*P. monodon*	Y	2.39	79	44,862	31,640	62.5/27.1	Illumina + PacBio + Chicago + HiC	2021	[10]
*P. monodon*	N	1.89	78	496	25, 809	61.8/30	Illumina + PacBio + 10×Genomics + HiC	2022	[76]
*M. japonicus*	N	1.70	113.13	235	26,381	49.76/27.44	Illumina + PacBio + HiC	2021	[80]
*M. japonicus*	Y	1.54	229.97	38,270	24,317	61.56/43.13	Illumina + PacBio + HiC	2022	[79]
*F. indicus*	Y	1.93	1,462,103	34,406	28,720	49.31/31.99	Illumina + PacBio + HiC	2022	[78]

## Data Availability

Not applicable.
